# Effects of Fusion between Tactile and Proprioceptive Inputs on Tactile Perception

**DOI:** 10.1371/journal.pone.0018073

**Published:** 2011-03-25

**Authors:** Jay P. Warren, Marco Santello, Stephen I. Helms Tillery

**Affiliations:** 1 School of Biological and Health System Engineering, Arizona State University, Tempe, Arizona, United States of America; 2 Psychology Department, Arizona State University, Tempe, Arizona, United States of America; The University of Western Ontario, Canada

## Abstract

Tactile perception is typically considered the result of cortical interpretation of afferent signals from a network of mechanical sensors underneath the skin. Yet, tactile illusion studies suggest that tactile perception can be elicited without afferent signals from mechanoceptors. Therefore, the extent that tactile perception arises from isomorphic mapping of tactile afferents onto the somatosensory cortex remains controversial. We tested whether isomorphic mapping of tactile afferent fibers onto the cortex leads directly to tactile perception by examining whether it is independent from proprioceptive input by evaluating the impact of different hand postures on the perception of a tactile illusion across fingertips. Using the Cutaneous Rabbit Effect, a well studied illusion evoking the perception that a stimulus occurs at a location where none has been delivered, we found that hand posture has a significant effect on the perception of the illusion across the fingertips. This finding emphasizes that tactile perception arises from integration of perceived mechanical and proprioceptive input and not purely from tactile interaction with the external environment.

## Introduction

Activity in the somatosensory cortex has been directly linked to conscious tactile perception [1], [2], [3], [4] in a similar manner to how the visual cortex is linked to visual perception [5], [6]. During the chain of events leading to interpretation of tactile information in S1, mechanical perturbation of the skin surface propagates through the epidermis to the four main types of mechanoceptors, each of which transduces a particular aspect of the perturbation into neural signals. These signals are relayed through the afferent peripheral neural network to the central nervous system. With few exceptions, these mechanical-tactile signals project to and are interpreted in the primary somatosensory cortex, S1 [7]. It may be that tactile perception is based on an isomorphic mapping between the skin and the S1 homunculus. However, the extent to which proprioception affects tactile perception is an issue still under debate in the literature [8], [9], [10], [11], [12]. Though the Cutaneous Rabbit Effect (CRE) has been studied across both continuous and non-continuous skin areas, posture plays an important role in eliciting the CRE across non-continuous skin regions in some situations (e.g. across crossed arms, [13]). However, the impact of posture on the CRE is less obvious across the fingertips [14] because the cortical region that is stimulated is located on one hemisphere and its somatotopic arrangement is isomorphic to continuous skin arrangements with no clear dependence on posture. Here we investigate the role that finger posture (proprioception) has on tactile perception of stimuli across the fingertips.

The CRE is a perceptual phenomenon where rapidly applied stimuli can induce the perception of a stimulus at a location where none was applied [1], [13], [15], [16], [17], [18], [19]. This illusory phenomenon has been identified in the auditory [20], [21], [22], [23], [24], [25], [26], visual [27], [28], and somatosensory systems [1], [13], [15], [16], [17], [18], [19]. Throughout its history several saltatory stimulation paradigms have been used to induce the CRE in the somatosensory system. One such paradigm, dubbed the reduced rabbit paradigm, utilizes three rapid stimulations presented at two physical locations, such that two stimuli are presented at the first location and a single stimulus at the second location. In this paradigm, the first ‘locator’ stimulus establishes the spatial (and perhaps temporal) origin. This stimulus is followed by the ‘attractee’ stimulus delivered to the same physical site as the ‘locator’ stimulus but shortly after. The third ‘attractor’ stimulus is presented at a different physical site than the ‘locator’ and ‘attractee’ stimuli a short time after the ‘attractee’ stimulus. The location and timing of the ‘attractor’ shifts the perceived location of the ‘attractee’ to a site closer to the ‘attractor's’ location [15], [16], [17], [18], [19]. In this paradigm, the perceived location of the ‘attractee’ can be manipulated using several factors including the rate of stimulation, location of the following ‘attractor’ stimuli, and overall stimulated body region [15], [16], [17], [29], [30], [31], [32], [33]. Recent studies have introduced an additional modification to the reduced rabbit paradigm. In the newer paradigm, the subject's attention is directed to a specific site between the ‘locator’ and ‘attractor’ sites and subjects are asked to indicate if they perceived this site as being stimulated during illusory trials [1], [13].

**Figure 1:**
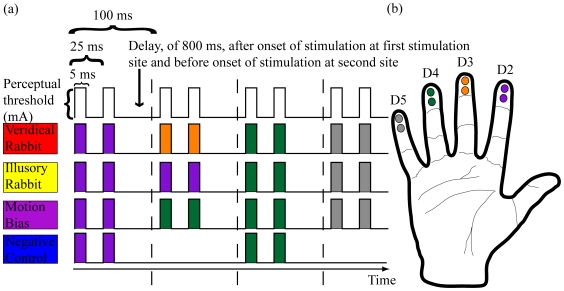
Schematics of stimulus sequences and electrode locations on fingertips. (a) Schematics of the four stimulus train types. All pulse widths are amplitude modulated to subject perceptual threshold for a particular stimulation site. Color is indicative of the stimulation site and/or train type. (b) Diagram of electrode locations on fingertips.

Recently we used the newer reduced rabbit paradigm to demonstrate that the CRE could be induced across the fingertips with electrotactile stimuli [14]. By applying a train of electrotactile pulses to the index, index, ring, and little fingertips (the *Illusory Rabbit Train*, Fig. 1), while all the fingers were extended (Fig. 1) we were able to induce the perception that the middle fingertip was stimulated though it received no stimuli under this train. To determine that the CRE was responsible for mislocalization of the stimulus onto the middle fingertip, and not another illusory effect or error type, we compared the Illusory Rabbit to another similar stimulus train (*Motion Bias Train*, Fig. 1). The Illusory Rabbit and Motion Bias Trains are both prone to a perceptual bias due to the anticipated velocity of the stimuli and stimulation of the sites surrounding the illusory site (here the middle fingertip). However, only the Illusory Rabbit Train should elicit the CRE due to perceptual length contraction [34]. Therefore if the CRE is elicited the Illusory Rabbit Train needs to have a higher mislocalization rate onto the illusory site, the middle fingertip, than the Motion Bias Train. We reported that the Illusory Rabbit Train had a higher mislocalization rate onto the middle fingertip than the Motion Bias Train, demonstrating that the CRE was elicited [14]. Additionally a *Negative Control Train* (Fig. 1) was used to verify that further breaking up the timing between pulses did not influence the perceived location of the attractee stimuli due to either anticipated velocity of stimuli or perceptual time contraction.

A recent study, demonstrated that the illusory stimuli activate the same region within area 3b as analogous physical (not illusory) stimuli on the associated skin region [1]. Similar results have been shown for the fingertip representation using other tactile illusions [3], [35], [36], [37]. Area 3b of the somatosensory cortex processes input from mechanical tactile sensory receptors from all over the body [38] including the forearm, hand, and fingers [4], [39], [40], [41], [42], [43], and its constituent cells are known to have postural tuning [44], [45], [46], but the impact of this postural tuning within a hand on the tactile representation in area 3b and on conscious tactile perception is not known.

**Figure 2:**
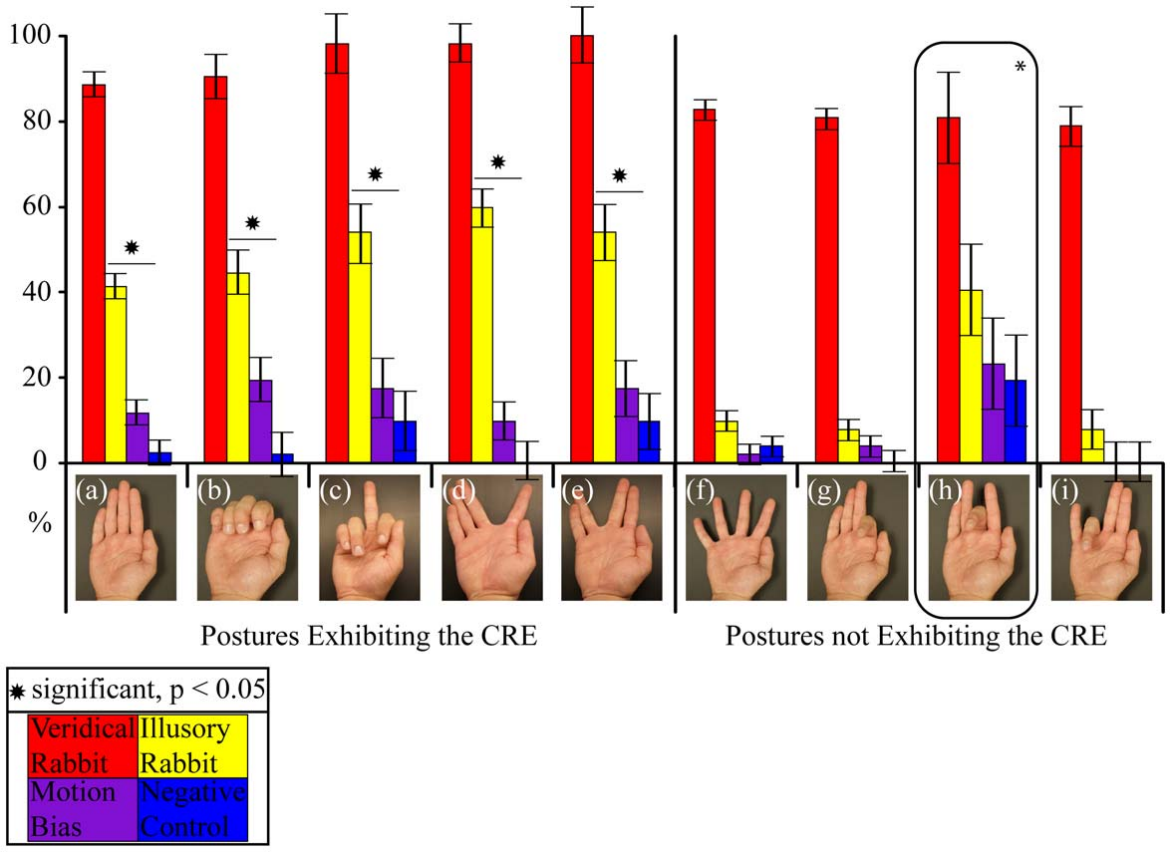
Percentage of trials where subjects indicated their middle fingertip was stimulated. Mean percentage of responses where subjects indicated that their middle fingertip had been stimulated; in response to, “Did the preceding stimulus train contain a stimulus on the middle fingertip?” Error bars indicate the standard error (s.e.m.) of each stimulus train in each posture. * indicate significant differences in subject perception of stimuli on the middle fingertip between the Illusory Rabbit and Motion Bias Trains for a particular posture as determined via Fisher LSD tests, α<0.05, (a) All-Adducted (*n* = 14), (b) All-Flexed (*n* = 4), (c) Middle-Extended (*n* = 4), (d) Index-Abducted (*n* = 4), (e) Vulcan (*n* = 4), (f) All-Abducted (*n* = 4), (g) Index-Flexed, (h) Middle-Flexed (*n* = 14), (i) Ring-Flexed (*n* = 4), where each *n* represents a subject response average of 13 trials per posture and stimulus train.

Here we demonstrate that hand posture does in fact play a significant role in the perception of the CRE across the fingertips. Using the techniques and stimulus trains we previously developed [14], [47], we tested the effect of nine hand postures on the perceptions of the four stimulus trains across the fingertips to determine how hand posture affected perception of this illusion. The nine postures were: (1) an *Adducted* posture (Figure 2(a)), where all fingers were extended and adducted, (2) an *All-Flexed* posture (Figure 2(b)) where all the fingertips were flexed and adducted, (3–5) postures where the *Index-*, *Middle-*, or *Ring-* fingertips were individually *Flexed* (Figures 2(h), (c), and (i) respectively) while the remainder of the fingers were positioned in a similar manner to the Adducted posture, (6) a *Middle-Extended* posture (Figure 2(d)) where the middle finger was extended while the other finger were flexed while adducted, (7) an *Index-Abducted* posture (Figure 2(e)) where the index finger was abducted away from the middle, ring, and little fingers which were touching, (8) a *Vulcan* posture (Figure 2(f)) where the index and middle fingers were touching but separated from the ring and little fingertips which were also touching and all extended, and (9) an *All-Abducted* posture (Figure 2(g)) where all of the fingers were extended and abducted from each other. We hypothesized that increasing the spatial distance between fingertips while they remained coplanar (All-Abducted, Index-Abducted, and Vulcan postures) would not change the mislocalization or illusory effect observed in the Adducted posture. However, we hypothesized that when one or more fingertips were not-coplanar with other fingertips (Index-Flexed, Middle-Flexed, Middle-Extended, and Ring-Flexed postures) the CRE seen in the Adducted posture would not be observed, as postural cues would eliminate the illusory effect.

## Results

We asked subjects to assume the Adducted and two or three of the other eight hand postures during testing. Subjects used a computer interface to administer their testing session; here the computer prompted the subjects that the next stimulus train was ready to be delivered, allowed the subjects to control when they were stimulated, and prompted them to record their response to each trial (see Methods). The subjects were instructed to answer, “Did the previous stimulus train contain a stimulus on the middle fingertip?” in response to each train of stimuli. To compare postures and stimulation trains across subjects we used a 2 factor factorial design. We found that the effect of hand posture was significant (ANOVA, α<0.05, p<0.0001). In this treatment of the data we considered all 14 of the subjects across all measured postures (9 postures in total). Subject responses for the 13 replicates of each stimulation train were averaged for each posture. The ANOVA table for this data can be found in Table 1.

**Table 1:** Analysis of Variance for 2-Factor Factorial Design.

			Mean	F	p-value
Source	Sum of Squares	df	Square	Value	Prob>F
A. Train Type	2.006 * 10^5^	3	66852.23	247.69	<0.0001
B. Posture	12019.72	8	1502.47	5.57	<0.0001
Interaction AB	9946.38	24	414.43	1.54	0.0644
Pure Error	39945.84	148	269.90		
Cor Total	2.625 * 10^5^	183			

Each subject (*n* = 14) was tested in either 3 or 4 postures. Every subject was tested in the Adducted posture as a preliminary check for influential subjects.

The data indicate that the subjects reported that their middle fingertip was stimulated under the Illusory Rabbit Train more often than the Motion Bias Train in five of the nine postures, but not for the Index-Flexed, Middle-Flexed, Ring-Flexed, and All-Abducted postures (Fig. 2, Individual Fisher LSD tests for each posture type, α<0.05, Table 2.). In the five postures where the Illusory Rabbit Train was reported to stimulate the middle fingertip more often than the Motion Bias Train, the Illusory Rabbit Train induced mislocalization onto the middle fingertip in more than 40% of trials, whereas in the other three of the other four postures the Illusory Rabbit Train induced mislocalization onto the middle fingertip in fewer than 10% of trials (Fig. 2). In the Middle-Flexed posture, one subject's data was found to be an outlier because of high Internally Studentized Residual values for the Motion Bias (3.244), Negative Control (4.055) Trains. Because this subject perceived that their middle fingertip was stimulated more often than the rest of the population for these trains it is not as clear whether the lack of difference between the Illusory and Motion Bias Trains for this posture was due to the influence of this subject or the posture. However, because the rate of mislocalization onto the middle fingertip was greater under the Illusory Rabbit Train than the Motion Bias Train in five postures, the increased rate of mislocalization can be attributed to the CRE and not other illusory effects in these postures. In the Index-Flexed, Middle-Flexed, Ring-Flexed, and All-Abducted postures, the Illusory Rabbit Train failed to increase the rate of mislocalization onto the middle fingertip above what was observed under the Motion Bias Trains indicating that the CRE was not induced in these postures. Together these findings indicate that hand posture does significantly affect the perception of the CRE across the fingertips.

**Table 2:** Fisher Least Significant Difference Table for Each Posture.

	Adducted	All-Flexed	Middle-Extended	Index-Abducted	Vulcan	Index-Flexed	Middle-Flexed	Ring-Flexed	All-Abducted
Veridical Rabbit vs. Illusory Rabbit Train	<0.0001	0.0008	0.0078	0.0040	0.0042	<0.0001	0.0828	<0.0001	<0.0001
Veridical Rabbit vs. Motion Bias Train	<0.0001	<0.0001	<0.0001	<0.0001	<0.0001	<0.0001	0.0192	<0.0001	<0.0001
Veridical Rabbit vs. Negative Control Train	<0.0001	<0.0001	<0.0001	<0.0001	<0.0001	<0.0001	0.0137	<0.0001	<0.0001
Illusory Rabbit vs. Motion Bias Train	<0.0001	0.0323	0.0219	<0.0001	0.0163	0.4537	0.4432	0.4222	0.1332
Illusory Rabbit vs. Negative Control Train	<0.0001	0.0015	0.0079	<0.0001	0.0055	0.1474	0.3412	0.4222	0.2503
Motion Bias vs. Negative Control Train	0.1168	0.1196	0.5900	0.3022	0.5676	0.4537	0.8600	1.0000	0.6944

Each cell contains the p-value for the Fischer Least Significant Difference Test.

## Discussion

### Posture dictates presence of the CRE across fingertips

We have quantified the effects of hand posture on the perception of the CRE across the fingertips. When subjects' attention was focused onto the presumed site of the mislocalization/illusion (the middle fingertip) we found that hand posture significantly affected the perception of a mislocalization onto this fingertip. Specifically we found that in five of the nine postures in this study that the CRE induced mislocalization of the attractee stimulus onto the middle fingertip. However, in the remaining four postures (Index-Flexed, Middle-Flexed, Ring-Flexed, and All-Abducted) the Illusory Rabbit and Motion Bias Trains induced a similar rate of mislocalization onto the middle fingertip, indicating that the CRE was not induced and only the Tau effect was needed to explain the mislocalization rate. Because of the differences in the presence of the CRE across these 9 postures, five inducing the CRE and four that were not susceptible to the CRE, we conclude that posture can significantly affect the ability to induce the CRE across fingertips.

### The Bayesian perceptual model for spatiotemporal illusions does not fully explain posture's effect on the CRE

Goldreich [34] proposed a Bayesian model that could be used to predict the likelihood of mislocalization onto a particular location based on three parameters: where attention was directed, the expected speed of stimulation, and direction of stimuli. The model predicts that the CRE and Tau effects would induce the expected mislocalization, onto the middle fingertip, in postures where motion of the illusory train of stimuli occurs along a line and the site of the mislocalization was equidistant from its two adjacently stimulated sites. The Tau effect occurs when the perceived distance between three consecutive stimuli is more correlated to the timing between the stimuli than the actual distance between them. This results in the perception that the stimuli closer together in time are separated by less distance than the ones that are stimulated further apart in time [34]. In the above experiment, three postures, the Adducted, All-Flexed, and All-Abducted, fit these criteria yet only two of them exhibited mislocalizations due to the CRE (Adducted and All-Flexed). The model further predicts that postures where the desired site of the mislocalization was not equidistant from the two stimulated sites should not exhibit the CRE or Tau effects as often as the postures that exhibit this arrangement, providing that the motion of the illusory train of stimuli occurs along a line. However, the two postures that fit these criteria (Index-Abducted and Vulcan) exhibited mislocalization of the illusory stimulus due to the CRE as often as the other (Adducted and All-Flexed) postures that exhibited the CRE. In the four (of the five) postures where the motion of the illusory train occurs along a line, we observed mislocalizations onto the middle fingertip attributable to the CRE suggesting that the spacing between the stimulated and unstimulated sites was not as important as the Bayesian model indicates.

There are two possible explanations for the failure of the Illusory Rabbit Train to induce the additional mislocalization of stimuli onto the middle fingertip in the All-Abducted posture, though the illusory and stimulated sites were collinear and equidistant from each other: (1) tactile interactions between stimulated fingertips and the presumed illusory fingertip aid in inducing the CRE, as in the Index-Abducted and Vulcan but not in the All-Abducted, and are absent in the All-Abducted; and/or (2) the increased space between the ‘cutaneous rabbit hops’ may lessen the CRE. Previous research has demonstrated that the illusory strength of the CRE (rate of mislocalization) decreases as saltatory area increases [17]. However, the present case is distinct because coordinates in the internal frame of reference remain the same (the stimulated skin sites), but the distance between these coordinates in the external reference frame change with hand posture. This suggests that the CRE is an illusory effect that takes into account the external frame of reference, in addition to the internal frame of reference and stimulated sites.

In postures where the perceived motion of the stimulus train would not occur along a line, the Bayesian model predicts that mislocalization due to the Tau or CRE should not occur (Index-Flexed, Middle-Flexed, Middle-Extended, and Ring-Flexed postures). Here, mislocalization of a stimulus onto the middle fingertip would force the perceptual ‘rabbit’ to jump out of its expected trajectory, along the line between the stimulated sites, and onto the non-collinear middle fingertip. Because this should result in a contradiction between the expectation of where the ‘rabbit’ should jump to (based on the direction of the stimuli) and the spatial location of the middle fingertip, the CRE and Tau effects are less likely to occur. However this is not what we observed. We found that mislocalization due to the CRE did not occur in the Index-, Middle-, and Ring-Flexed postures, but in the Middle-Extended posture the mislocalization attributable to the CRE was perceived at similar levels to those where the fingertips were collinear. The most likely explanation of this is that the collinearity of stimulated sites is more important than the collinearity of the expected trajectory of the illusory mislocalization, i.e. the posture of the stimulated sites are more important to determining whether or not illusory phenomena will influence the perception than the posture/position of the unstimulated (expected illusory) site. In the Middle-Flexed posture there was an overall high rate of mislocalization of stimuli onto the middle fingertip due to the Illusory Rabbit Train in comparison to the Index- and Ring-Flexed. Though there was an influential subject in the Middle-Flexed posture, the similar rates of mislocalization under Illusory Trains suggest that this posture may be more similar to the Middle-Extended posture than the Index- and Ring-Flexed postures. Together these data indicate postures where the stimulated sites are collinear are more likely to allow for mislocalization of stimulus location onto the unstimulated (expected illusory) site than postures where the stimulated sites are non-linear regardless of the unstimulated site's position relative to the stimulated sites.

Though evidence exists that the perceptual basis for the CRE is found in unimodal tactile maps located in area 3b of somatosensory cortex [1], the evidence here suggests that the perception of this illusory phenomenon must include information from cortical areas that receive significant postural or proprioceptive input. Currently area 3b of somatosensory cortex is not known to include significant amounts of postural or proprioceptive input [38], [44], [45], [46], [48], [49], [50], [51], [52] despite postural tuning of some 3b neurons [44], [46]. If tactile perception of these stimuli were to directly arise from the cortical information processed in area 3b of somatosensory cortex, changes in posture are therefore not likely to affect the perception of these illusory stimuli. However, our experiment demonstrated that changes in posture provide proprioceptive input that can either turn on or off the CRE. Therefore the perception of the tactile Cutaneous Rabbit and Tau Effects, and likely other tactile spatiotemporal illusions, should consider cortical processing from centers that include significant input from tactile, postural, and proprioceptive centers.

## Materials and Methods

### Human Subjects

Subjects (*n* = 14) participated in this study. Subjects were selected for participation from the greater Phoenix community if they were without history of neurological disease or current peripheral neurological injury (cut, burn, bruise, etc) that might affect their ability to perceive stimuli on their right hands. All subjects were familiar with experiencing electrotactile stimuli on their fingertips from prior experiences in this laboratory or others. However, none of the subjects were familiar with the CRE, the electrotactile stimulation trains, or the stimulation paradigm used in these experiments. Written informed consent documents were reviewed and signed by all participants before the experimental session began. These documents and procedures were previously reviewed and approved by the Institutional Review Board at Arizona State University, and were in accordance with the Declaration of Helsinki.

### Subject Electrode Interface

During the experiment, subjects were outfitted with a pair of electrodes centered on the volar aspect of their index, middle, ring, and little fingertips (Fig. 1). Each electrode pair consisted of two 3.2 mm diameter custom-made electrodes affixed to non-distensible, clear tape in a similar fashion to our prior studies [47], [53]. The electrodes had a center-to-center spacing of 10 mm along the long axis of the fingertip. Each electrode had electrode cream (Genuine Grass EC2™ Electrode Cream, Astro-Med, Inc., Grass Instrument Division, W. Warwick, RI, USA) carefully applied onto it in order to lower the skin impedance and provide a uniform interface between the electrode and fingertip.

### Electrical Stimulation Setup

Electrical stimuli, anodic half-rectified square waves, were delivered to the skin surface via the custom made electrode pairs described above [53]. The electrical current was provided to the electrodes via constant current linear isolators (4, DLS100s, World Precision Instruments, Sarasota, FL, USA) that were triggered by a digital stimulator (DS8000 Digital Stimulator, World Precision Instruments). We used the method of limits [54] to determine the perceptual thresholds, the minimum current necessary to be detectable in 10 consecutive stimulations. We then used those stimulation levels, as determined for each digit, in the experiments. The stimulus trains used in this experiment were comprised of 2 pulses per digit delivered 25 ms from onset to onset. A delay of 800 ms between the onset of the locator and attractee stimuli was used to separate the temporal and spatial influences of the locator stimulus (Fig. 1). The onset of the final two stimuli was 100 ms after the onset of the second (attractee) stimulus. The digital stimulator was controlled from a desktop computer through Labview® software (Labview 8.5, National Instruments, Austin, TX, USA) created specifically for this purpose.

### Electrical Stimulus Trains

Labview® software was programmed to send one of the four stimulus trains (Veridical Rabbit, Illusory Rabbit, Motion Bias, or Negative Control Trains) at random to the digital stimulator and onto the subject. Veridical Rabbit Trains consisted of two pulses sent to the index, middle, ring, and little fingertip, ‘D2-D3-D4-D5’, at the timing described above. Illusory Rabbit Trains consisted of stimuli at ‘D2-D2-D4-D5’. The Motion Bias Trains consisted of stimuli delivered to ‘D2-D4-D4-D5’and the Negative Control Trains consisted of stimuli delivered to D2 at 0 ms and D4 at 900ms (Fig. 1).

### Subject Setup

During setup and experimental trials, subjects were seated with their elbow bent and arm comfortably resting on a desk in front of them. Subjects were instructed that they could position their forearm and hand in front of them in a comfortable position. Throughout the trial subjects had full view of their hand and fingertips and a reference figure noting the names of the fingertip sites. The desk contained a computer monitor and a mouse, which the subject used to send the stimuli and record their responses. The monitor displayed a Labview® user interface that prompted subjects with appropriate controls to enter their responses, proceed to the next trial and send the next stimulus train. After clicking the button to deliver the next stimulus train, the program would select a train from a randomized rubric and send the appropriately timed train information to the digital stimulator. Once the subject received the electrical stimuli, the proper response types would appear on the screen allowing the subject recorded their response and then continue on to the next frame, repeating the process.

### Experimental Design

A two-factor factorial design with factors, stimulus train type (Veridical Rabbit, Illusory Rabbit, Motion Bias, and Negative Control Trains) and posture (Adducted, All-Flexed, Index-Flexed, Middle-Flexed, Middle-Extended, Ring-Flexed, Index-Abducted, Vulcan, and All-Abducted) was chosen to design and analyze the data for this experiment. A factorial design was chosen instead of a repeated measures design because there were few differences in experience, training, or background of the subjects in this experiment which makes it unlikely that real differences between treatments would be confounded with inter-subject variance other than their perception of the CRE, which could not be separated by a repeated measure design. The experimental design was created and analyzed using Design-Expert® (Design-Expert v 7.0, Stat-Ease Inc., Minneapolis, MN). Overall, 14 subjects were used in this experiment, 10 of the subjects performed three experimental postures while four subjects performed four experimental postures. Each subject performed the Adducted posture to establish a baseline for their perception of the CRE across their fingertips.

### Factors

#### Stimulus Trains

The four stimulus train factor levels were the train types previously described: Veridical Rabbit, Illusory Rabbit, Motion Bias, and Negative Control Trains.

#### Postures

There were 9 factor levels or hand postures considered in this experiment, Adducted, All-Flexed, Index-Flexed, Middle-Flexed, Middle-Extended (all others flexed), Ring-Flexed, Index-Abducted, Vulcan (index and middle abducted away from the ring and little fingers, each pair touching), and All-Abducted (from each other, Fig. 1). In the non-flexed postures the subject's fingers are extended at each finger joint. In the All-Flexed and Middle-Extended posture each of the flexed fingers were flexed at the metacarpo-phalangeal (MCP), proximal interphalangeal (PIP), and distal interphalangeal (DIP) joints. Because assuming some of these postures was difficult for some, some minor digit flexion of the other digits was allowed, providing that this flexion was less than or equal to 30 degrees (visually estimated) from the adducted posture.

### Experimental Protocol

Each experimental session began by placing the electrode pairs onto the fingertips of the right hand and determining their perceptual thresholds. Seated at the desk and in front of the computer, each subject was exposed to the Veridical Rabbit Train to ensure that the electrical stimuli on each fingertip were approximately equal in perceptual intensity. Each subject's experimental session contained the adducted posture and either two or three other randomly assigned postures. Subjects were asked to assume a particular hand posture and respond to a particular question during each block of 52 trials. Inside a block, the stimulation trains were mixed randomly so that each was replicated 13 times. The order these blocks were presented was randomized so that each subject had to perform 3 or 4 blocks (one for each posture) to complete the experiment, totaling either 156 or 208 total stimulus trains per subject. Most experimental sessions took between 45 and 60 minutes to complete once electrodes setup began.

### Statistical Analysis

Data analysis was performed using Design-Expert® using the general factorial design described above using the percentage of correct subject responses to “Did the preceding stimulus train contain a stimulus on the middle fingertip, D3?” for analysis. Analysis Of Variance (ANOVA) statistics were computed for the two-factor factorial design (posture and stimulus train type). Additionally ANOVAs and Fisher's Least Significant Difference (LSD) tests were performed on each posture to compare between the stimulus train types within a posture because there were significant differences between the postures. Detailed explanation of factorial analysis can be found in, Design and Analysis of Experiments [55].
